# Lonnie O'Neal Ingram (1947-2020)—Dedication and determination

**DOI:** 10.1093/jimb/kuac004

**Published:** 2022-03-15

**Authors:** K T Shanmugam

**Affiliations:** Department of Microbiology and Cell Science, University of Florida, Gainesville, FL 32611, USA

Dr. Lonnie O'Neal Ingram, a brilliant microbial physiologist, metabolic engineer, and synthetic biologist, passed away on June 25, 2020, at the age of 72 in Charleston, South Carolina. He graduated from the University of South Carolina in 1969 and just after two years received his Ph.D. degree in 1971 from the University of Texas. After a short postdoctoral study at the Oak Ridge National Laboratory, Dr. Ingram joined the Department of Microbiology and Cell Science at the University of Florida in 1972, where he spent a distinguished academic career until his retirement in 2017. He was the founding director of the Florida Center for Renewable Chemicals and Fuels at the University of Florida, from 1998 until his retirement.

Looking back at his career, ethanol played a significant part in his academic life, initially as a tool to study membrane synthesis and architecture and later maximizing its production as a transportation fuel. Developing microbial biocatalysts for production of ethanol as a fuel consumed most of his life. He worked toward this goal tirelessly. Those of us who knew him well can recall his almost 24/7 dedication towards this goal. When the State of Florida granted the funds for a pilot-scale cellulosic ethanol plant toward developing a cost-effective biorefinery, he became an engineer and oversaw the construction of the plant. Since the plant was located at Perry, Florida, this required a round-trip travel of about 180 miles two to three times a week. The integrated process for converting biomass to fuel ethanol at this pilot plant was his own design upgraded from a unit operations plant he designed and ran at the main campus of the University of Florida. This was the first integrated pilot plant for cellulosic ethanol production associated with a university anywhere in the world and this plant did deliver a simple process for conversion of biomass, in this case, sugarcane and sorghum bagasse, to ethanol due to Neal's dedication and determination.

**Figure:**
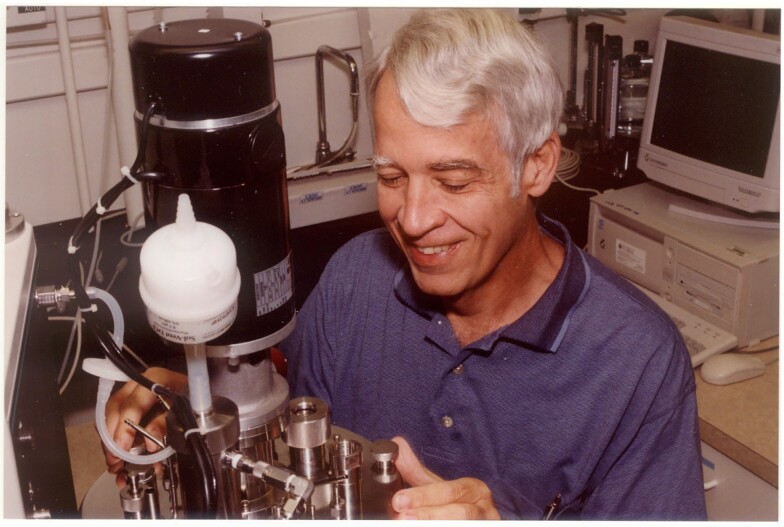


Major advances in science are made by unique individuals taking steps that are usually ahead of their time. The pioneering development of ethanologenic *Escherichia coli* by introducing pyruvate decarboxylase and alcohol dehydrogenase genes from *Zymomonas mobilis* into *E. coli* is a significant philosophical step in metabolic engineering. This paradigm shift is an early version of synthetic biology that was recognized by the granting of the landmark patent, number 5 000 000, by the US Department of Commerce. This was followed by several other patents for engineered microorganisms for production of various chemicals. The accolades he received include commendations by the State of Florida Senate and House, a Distinguished Service Award from the US Department of Agriculture (the highest presented for research), and election to the National Academy of Sciences (2001) and US National Academy of Inventors (2013).

Another case in point was “Zoolibraries”. In one of our conversations, he raised the possibility of cloning genes for a needed trait directly from the environment without an intermediate step of isolating and culturing the needed microorganism as a source of the gene. A manuscript describing this technology was rejected even by journals dealing with microbial biotechnology at that time as trivial. As history reveals, several biotech companies since and now rely on direct cloning from the environment as an asset. This shows that Neal was able to see ahead of time into what the future holds in industrial microbiology and be there first.

Although Lonnie Ingram developed several microbial biocatalysts and processes, he always thought of himself as a teacher and mentor and not as a businessman. A large team of students, postdoctoral associates, and visitors were always working in his lab. The scientists he trained are around the globe and fondly remember him and carry on the tradition established at his lab. For some of these students, building inexpensive pH-controlled fermentation units was a rite of passage as they entered his laboratory and these units are still in their labs working hard toward adaptive metabolic evolution of microorganisms.

His kind nature was to help others as best he could and try to bring out the best in everyone. He collaborated with most of the faculty members in his home department at various times and with several others within and outside the USA. At work, he was an inspiration and guiding light to his peers. He is missed by all who knew him.

K.T. Shanmugam, a friend and colleague

